# Integrated Screening Cascades for Ion-Channel Drug Discovery: Linking Structure, Electrophysiology, Safety Pharmacology, and Human-Relevant Models

**DOI:** 10.3390/ijms27135774

**Published:** 2026-06-26

**Authors:** Yohan Seo

**Affiliations:** Department of Physiology, Dongguk University College of Medicine, Gyeongju 38066, Republic of Korea; ddukdae12@dongguk.ac.kr or ddukdae12@gmail.com; Tel.: +82-54-770-2416

**Keywords:** ion channels, high-throughput screening, automated patch clamp, cancer, inflammation, organoids, microphysiological systems, safety pharmacology, artificial intelligence, state-dependent pharmacology

## Abstract

Ion channels are validated drug targets, but they remain difficult to study as their pharmacology is influenced by rapid gating, conformational state transitions, cell-type-specific expression, and narrow safety margins. Recent advances in cryo-electron microscopy, structure-based in silico screening, machine-learning-guided prioritization, optical high-throughput screening, automated patch-clamp electrophysiology, and human-relevant organoid or microphysiological system (MPS) models are transforming this field. In this expanded review, we examine how these modalities can be integrated into a hybrid discovery pipeline that begins with computational triage, proceeds through scalable functional screening and state-aware electrophysiological validation, and concludes with multi-channel safety de-risking and translational analysis in complex human models. We also discuss disease-associated channel remodeling in cancer and inflammatory disorders, with an emphasis on transient receptor potential channels, voltage-gated potassium channel 1.3 (Kv1.3), Piezo channels, transmembrane protein 16A/anoctamin-1 (TMEM16A/ANO1), chloride channels, and proarrhythmic safety risks. Additionally, we highlight unresolved challenges, including bias in artificial intelligence models, incomplete conformational sampling, assay interference, organoid heterogeneity, and regulatory acceptance of MPS platforms. This review proposes a staged decision framework in which computational prioritization, scalable functional screening, direct electrophysiological confirmation, safety pharmacology, DMPK assessment, and disease-relevant human models serve as complementary filters rather than competing platforms for the identification of selective and translatable ion-channel therapeutics.

## 1. Introduction

Ion channels are among the largest and most pharmacologically tractable classes of membrane proteins. They translate chemical, electrical, mechanical, and osmotic cues into changes in membrane potential, ionic flux, and downstream cellular behavior [1,2,3]. Given that these processes are positioned at the interface between extracellular stimuli and intracellular pathways, channel modulators can precisely influence excitability, secretion, immune activation, epithelial transport, and cell motility.

The therapeutic potential of ion channels is further supported by broader drug–target analyses. Classical surveys have emphasized membrane proteins, including ion channels, as major components of clinically actionable biology [4,5]. Subsequent focused reviews argued that ion channels could become a major class of small-molecule targets if screening platforms and safety profiling advanced sufficiently to address their biophysical complexity [6,7]. Accordingly, modern ion-channel discovery now extends beyond neurophysiology and cardiology into oncology, immunology, epithelial biology, and mechanobiology.

The same features that make ion channels attractive drug targets also make them technically difficult to investigate. Channel activity is not a static binding event but a time-dependent conductance process shaped by voltage, ligand binding, lipid environment, accessory proteins, gating history, and cellular context [8,9,10,11]. Therefore, a compound that appears inactive under one protocol may prove potent under another that favors an open, inactivated, or desensitized state. This protocol dependence is a central reason ion-channel screening cannot rely solely on simple endpoint assays.

Structural biology has transformed this challenge into an opportunity for discovery. Structures of voltage-gated sodium and potassium channels have revealed how pores, selectivity filters, voltage sensors, and intracellular gates cooperate to regulate ion flow [12,13,14,15]. Subsequent studies linked these structural insights to channelopathies, subtype selectivity, and mechanisms of drug binding [16,17,18,19]. A modern review of ion-channel discovery must therefore integrate target biology, conformational pharmacology, and translational validation.

Cancer and inflammatory disorders illustrate why this integrated view matters. Tumors remodel ion-channel expression and activity in response to hypoxia, acidosis, growth factors, extracellular matrix stiffness, and treatment pressure [20,21,22]. These changes can promote calcium-dependent transcription, proliferation, migration, invasion, and survival. In parallel, immune and epithelial cells use ion channels to regulate activation thresholds, cytokine release, barrier function, and tissue repair [23,24]. The key pharmacological question is therefore not only whether a compound blocks a recombinant channel, but whether it alters disease-relevant channel behavior in a setting that resembles the human lesion.

Ion-channel pharmacology has also become a platform challenge. Optical high-throughput screening (HTS) can provide scalable functional screening, automated patch-clamp (APC) can verify ionic currents and state dependence, and computational methods can enrich libraries before experimental testing [25,26,27,28,29]. These approaches complement rather than replace each other. This review argues that selective, translatable ion-channel therapeutics require a staged evidence framework that integrates throughput, mechanism, safety, and human-relevant disease models.

Unlike previous reviews that have focused primarily on individual ion-channel families, electrophysiological methods, disease-specific channel biology, or cardiac safety assessment, this review proposes an integrated decision architecture for ion-channel drug discovery. The central premise is that candidate progression should be based on staged evidence across complementary dimensions: structural and computational plausibility, scalable functional activity, direct electrophysiological confirmation, safety and selectivity de-risking, DMPK and exposure assessment, and human-context translational validation. This framework is intended to reduce assay-driven false positives, distinguish direct channel modulation from broader cellular effects, and improve the translational relevance of ion-channel lead selection.

## 2. Why Ion Channels Remain Difficult Drug Targets

### 2.1. Conformational Dynamics and State Dependence

The first barrier is conformational dynamics. Ion channels transition among resting, open, inactivated, desensitized, ligand-bound, lipid-bound, and drug-bound states. These transitions occur over timescales from microseconds to minutes and determine whether a ligand can access its binding site or stabilize a specific gating state [8,9,10,11]. Consequently, equilibrium potency values alone rarely capture the full pharmacology of a channel modulator.

Use dependence and state dependence are especially important in drug discovery. A use-dependent blocker may preferentially inhibit hyperactive cells, as repeated depolarization increases occupancy of open or inactivated states [30,31]. This may confer disease-state selectivity; however, assays restricted to a single time point under a uniform stimulus may fail to detect it. Conversely, compounds that perturb membranes or intracellular calcium stores can generate optical signals that mimic channel block without directly acting on channel pores [32,33].

### 2.2. Subtype Selectivity and Channel-Family Conservation

The second major barrier is subtype selectivity. Closely related channel paralogs often preserve conserved pore-lining residues, voltage-sensing motifs, and ion-conduction pathways. Potassium channels exemplify this challenge: related Kv family members may share a highly similar pore architecture yet differ substantially in tissue distribution, auxiliary subunit composition, and functional roles in immune regulation or cellular proliferation [7,15]. Accordingly, drug-discovery efforts must assess not only potency at the intended target but also selectivity across closely related members of the channel family.

Transient receptor potential (TRP) channels, sodium channels, calcium-activated chloride channels, and mechanosensitive channels present analogous selectivity challenges. TRP channels function as cellular sensors of temperature, mechanical stress, irritants, osmotic pressure, and inflammatory signaling [34,35,36,37,38]. Although this functional diversity creates substantial therapeutic opportunity, inadequate selectivity may perturb multiple sensory or inflammatory pathways simultaneously. In oncology, this challenge is further compounded by channel expression patterns that vary across tumor subtypes, stages, microenvironmental niches, and prior treatment histories [20,21,22,39,40,41,42].

### 2.3. Safety Liabilities and Proarrhythmic Risk

The third barrier is safety. Blocking hERG (human Ether-à-go-go-Related Gene)/Kv11.1 can delay ventricular repolarization and increase proarrhythmic risk, a liability that has affected multiple drug classes [33,43]. Given that many ion-channel-active chemotypes are sufficiently hydrophobic and basic to interact with cardiac channels, early hERG and broader multi-channel profiling should be treated as essential parts of discovery, not as late-stage checks.

Modern cardiac safety assessment extends beyond a hERG-centric paradigm. The CiPA framework integrates multi-ion-channel data, in silico models of human ventricular action potential, and human induced pluripotent stem cell-derived cardiomyocytes [44,45,46,47]. This approach is important for ion-channel programs because proarrhythmia is shaped not only by hERG inhibition, but by the integrated balance of inward and outward currents, repolarization reserve, drug exposure, and clinical context [48,49,50,51,52,53,54].

### 2.4. Disease-Context Translation

The fourth barrier is disease-context translation. Recombinant systems are indispensable for clean mechanistic studies, but they often omit the membrane microenvironment, accessory proteins, paracrine cues, matrix tension, inflammatory priming, and heterocellular interactions that shape channel behavior in disease [55,56]. As a result, a blocker that appears potent in a simplified cell line may fail in organoids if the relevant channel is confined to a differentiated or polarized compartment that the screening system does not reproduce.

Human organoids and MPS platforms partially bridge this gap by preserving tissue architecture, donor variability, flow, mechanical forces, and tissue–tissue interfaces [57,58,59,60,61]. They are most informative after the mechanism is established, because in complex models, a phenotype may reflect direct channel modulation, cytotoxicity, altered differentiation, or indirect signaling. For ion channels, translational validation should therefore be hypothesis-driven rather than purely phenotypic.

A general principle across the cascade is that direct channel modulation must be distinguished from indirect phenotypic effects. A compound may reduce proliferation, migration, invasion, cytokine release, barrier dysfunction, or organoid growth without directly modulating the intended ion channel. Such effects may arise from cytotoxicity, altered metabolism, upstream receptor inhibition, membrane perturbation, changes in differentiation state, or off-target signaling. Therefore, disease-context phenotypes should be interpreted together with direct current measurements, orthogonal flux or voltage assays, target expression, genetic perturbation, rescue experiments, and exposure controls. This distinction is particularly important in cancer and inflammatory models, where cellular phenotypes are influenced by multicellular signaling and microenvironmental stress in addition to channel conductance.

A second general principle is that acute modulation of channel conductance should be separated from longer-term channel remodeling. Acute inhibition refers to rapid changes in ionic current that occur before detectable changes in channel abundance, localization, trafficking, or cell state. In contrast, channel remodeling may involve altered transcription, translation, membrane trafficking, degradation, subcellular localization, accessory-subunit composition, or differentiation-dependent expression. These mechanisms may produce similar phenotypic outcomes but require different assays and different medicinal chemistry decisions. For this reason, ion-channel discovery programs should specify whether a candidate acts as an acute blocker, gating modifier, allosteric modulator, expression-level regulator, trafficking corrector, or broader cellular perturbant.

## 3. Screening Modalities: Throughput, Mechanism, and Translational Value

### 3.1. Optical HTS as a Scalable Discovery Layer

Optical HTS remains an important component of contemporary discovery workflows because it enables the interrogation of large chemical libraries through calcium-flux assays, membrane-potential dyes, ion-sensitive probes, fluorescence resonance energy transfer, and high-content imaging [26,27]. These assay formats are readily miniaturized and can be implemented in primary cells as well as disease-relevant phenotypic models. Their principal advantage lies in throughput, permitting broad exploration of chemical diversity before progression to more resource-intensive electrophysiological studies.

Nevertheless, data derived from optical HTS require careful interpretation. Fluorescent dyes may be influenced by compound autofluorescence, quenching, dye loading, transporter activity, cytotoxicity, and alterations in upstream receptors or intracellular stores [62,63,64,65]. When such findings are used to support a discovery program, reporting should include assay-quality metrics such as Z-prime, signal window, replicate concordance, and concentration–response reproducibility [63,64]. Accordingly, optical HTS is most appropriately used to identify candidate compounds rather than to establish mechanisms. In optical HTS, chemical interference should be treated as a major source of false-positive activity. These liabilities are particularly relevant for ion-channel assays because changes in membrane potential, intracellular calcium, chloride flux, or fluorescence intensity can arise from indirect effects rather than direct channel modulation. Therefore, primary optical hits should be triaged using orthogonal counter-screens, cytotoxicity normalization, dye-free or label-free assays, detergent-sensitivity or aggregation controls when appropriate, and direct electrophysiological confirmation before mechanistic claims are made.

### 3.2. Automated Patch Clamp as a Mechanistic Confirmation Layer

APC is a mechanistic cornerstone of ion-channel pharmacology because it enables direct measurement of ionic current rather than reliance on a surrogate signal [25,26]. Contemporary platforms support voltage-clamp protocols, ligand application, concentration–response analysis, recovery-from-inactivation studies, and use-dependence testing at a scale not attainable with conventional manual patch clamp [27,28,29]. These capabilities are critical for distinguishing pore block, allosteric modulation, kinetic trapping, and apparent inhibition attributable to cytotoxicity or membrane disruption.

The placement of APC within the discovery workflow should be determined by the pharmacological properties of the target. For targets whose disease relevance depends on firing frequency, depolarization history, mechanical stimulation, or calcium-dependent activation, APC should be incorporated earlier in the cascade. When the primary screen is highly reliable and supported by orthogonal counter-screens, APC may serve primarily as a confirmatory and SAR-guiding step. In either context, APC is the stage at which a chemical hit becomes a mechanistically interpretable channel modulator. Although APC provides direct current measurements, its data should be interpreted in light of platform-specific limitations. Seal quality, access resistance, series-resistance error, voltage-control limitations, cell-line background currents, intracellular dialysis, current rundown, temperature, and compound equilibration time can all influence apparent potency and kinetics [25,26,27,28,29]. These issues are especially important for rapidly gating sodium channels, state-dependent blockers, and channels with substantial rundown during repeated stimulation. Consequently, APC-based validation should report not only IC50 values, but also the voltage protocol, holding potential, pulse frequency, exposure duration, washout behavior, recording acceptance criteria, and whether the observed inhibition is reversible, state-dependent, use-dependent, or associated with nonspecific deterioration of cell health.

### 3.3. Structure-Enabled and AI-Assisted Triage

Structure-enabled and AI-assisted triage can substantially streamline discovery before wet-lab testing. Resources such as AlphaFold, RoseTTAFold, molecular docking, ChEMBL, DrugBank, decoy libraries, and ADMET prediction platforms can be used to prioritize compounds, identify liabilities, and generate structure–function hypotheses [66,67,68,69,70,71,72,73,74,75,76,77,78,79,80]. These approaches are particularly valuable when experimental structures are incomplete, virtual libraries are large, or medicinal chemistry efforts require early filters for selectivity and developability.

However, computational predictions should guide prioritization rather than serve as proof. Membrane proteins remain underrepresented in many training sets, binding sites may be state-dependent, and lipids or accessory subunits can create determinants that static structures do not capture [66,67]. In ion-channel discovery, an AI-prioritized compound becomes compelling only when predicted activity is supported by functional current modulation, kinetic behavior, and selectivity data. For structure-enabled discovery, static docking should be interpreted as hypothesis generation rather than mechanistic proof. Ion channels occupy multiple conformational states, and ligand access may depend on the voltage-sensor position, pore hydration, intracellular gates, lipid environment, membrane potential, and auxiliary subunits [66,67,68,69,70,71,72,73,74,75,76,77,78,79,80]. Therefore, state-aware ensemble docking, molecular dynamics simulations, and, where feasible, free-energy calculations can improve prioritization by sampling conformations that are not captured by a single cryo-EM structure or predicted model [81]. Computational predictions become persuasive only when supported by experimental validation, including mutational sensitivity analyses, electrophysiological characterization, and selectivity profiling.

### 3.4. Organoids and MPS Platforms as Translational Filters

Organoids and MPS models are increasingly incorporated at later stages of the discovery cascade. Patient-derived organoids can retain features of tumor heterogeneity and treatment response, thereby enabling evaluation of candidate compounds in models that more closely approximate clinical biology [60,61,82,83,84]. MPS platforms introduce parameters such as flow, mechanical stretch, compartmentalization, and organ–organ communication, which are particularly relevant for channels regulated by shear stress, barrier integrity, or tissue mechanics [85,86,87,88,89,90,91,92].

These models do not supplant electrophysiological analysis; rather, they assess whether a mechanistically validated modulator retains activity within a human-like context. Co-culture and three-dimensional systems may reveal effects of channel modulation on stromal signaling, epithelial barrier integrity, immune activation, or drug penetration [93,94,95]. Accordingly, a negative result in an organoid or MPS assay should prompt mechanistic follow-up rather than immediate abandonment, as such findings may reflect compound exposure, differentiation state, matrix composition, or endpoint selection rather than absence of biological activity. The complementary strengths and limitations of these modalities are summarized in Table 1.

## 4. Disease-Linked Channel Remodeling in Cancer and Inflammatory Disease

### 4.1. Cancer-Associated Channel Remodeling

Cancer-associated channel remodeling is a network-level process rather than a single-target event. TRP channels can regulate calcium entry, stress sensing, migration, invasion, angiogenesis, and drug response [20,34,35,36,37]. Potassium channels shape membrane potential, calcium signaling, proliferation, and immune interactions [21,22]. Sodium channels and other conductance pathways can also promote invasive behavior and metastatic traits in selected tumor settings [39,40].

The concept of “oncochannelopathy” posits that malignant phenotypes may arise from altered channel expression, subcellular localization, or gating behavior, rather than exclusively from canonical kinase signaling [20]. Ion channels also interact with transporters, cytoskeletal regulators, cell volume control pathways, and the molecular machinery that governs cell migration [41,42]. Accordingly, cancer-oriented ion-channel screening strategies should integrate direct measurements of channel activity with disease-relevant phenotypic endpoints, including migration, invasion, stress survival, and pathway modulation.

Piezo channels further underscore the importance of mechanobiology in cancer. Piezo1 and Piezo2 are mechanically activated cation channels that connect membrane tension and tissue-level forces to calcium-dependent signaling [96,97,98]. Piezo1 has established roles in vascular development and mechanotransduction, and chemical probes such as Yoda1 have facilitated functional interrogation of Piezo1-dependent responses [99,100,101,102]. In tumors, these channels may link extracellular-matrix stiffness, stromal tension, and invasive behavior to a pharmacologically tractable conductance pathway.

From an assay-design perspective, cancer-associated channel remodeling means that recombinant current inhibition is necessary but not sufficient. TRP- or potassium-channel activity may need to be tested under inflammatory priming, hypoxia, acidosis, or growth-factor stimulation. Mechanosensitive channels such as Piezo1 require stimulation protocols that reproduce membrane tension, matrix stiffness, shear stress, or stretch. Sodium-channel- or chloride-channel-associated invasion phenotypes should be linked to direct current modulation, genetic knockdown or rescue, and non-cytotoxic concentration ranges. Thus, cancer-oriented ion-channel screening should combine direct electrophysiological or flux-based readouts with phenotypic assays for migration, invasion, survival, and pathway modulation, while controlling for nonspecific cytotoxicity and indirect pathway effects.

### 4.2. Immune and Inflammatory Remodeling of Ion-Channel Function

Inflammatory disorders are characterized by remodeling of the channelome across T cells, macrophages, dendritic cells, epithelial cells, sensory neurons, and stromal populations. In immune cells, potassium and calcium channels are required to maintain membrane potential, sustain calcium influx, and regulate cytokine production [23,24]. Kv1.3 is a prototypical therapeutic target because effector memory T cells depend on potassium conductance to maintain the electrical driving force required for calcium entry during activation [103,104].

Kv1.3 pharmacology further illustrates the extent to which selectivity and therapeutic modality determine translational feasibility. Peptide blockers and venom-derived ligands may achieve high potency and selectivity, yet their development can be constrained by delivery limitations and immunogenicity [105,106]. Consequently, small-molecule programs have emphasized the need to balance potency, selectivity, pharmacokinetics, and safety [107]. For inflammatory models, Kv1.3 should be considered within a broader immune-channel network rather than as an isolated conductance pathway. Kv1.3 and KCa3.1 both contribute to the maintenance of membrane potential required for sustained calcium entry during lymphocyte activation, and their relative importance may vary according to T-cell differentiation state, activation history, and disease context [23,24,103,104,108]. This distinction is pharmacologically important because a compound that appears effective in one immune-cell state may be less active in another if compensatory potassium conductances preserve calcium signaling. Accordingly, immune-cell screening should include activation-state-defined assays, relevant cytokine readouts, and selectivity profiling across functionally related potassium channels. More broadly, therapeutic strategies directed at inflammatory ion-channel targets must align biological relevance with a practically deployable modality.

TRP and Piezo channels provide complementary mechanistic links among inflammation, pain, mechanosensation, and tissue remodeling [34,96]. In epithelial inflammatory states, ion channels can influence secretion, barrier integrity, mucosal hydration, and tissue repair. Given that these processes are highly context-dependent, primary epithelial cultures, organoids, and inflammatory co-culture systems can provide substantial translational value once the underlying electrophysiological mechanisms have been established.

For inflammatory disease screening, assay design should define the immune-cell state being modeled. Resting, recently activated, chronically stimulated, effector memory, and tissue-resident immune cells may differ in channel expression, membrane potential, calcium dependence, cytokine output, and sensitivity to Kv1.3 or KCa3.1 modulation. Functional screening should therefore combine current or membrane-potential measurements with activation-state markers, cytokine readouts, viability controls, and selectivity profiling across related potassium and calcium channels. In epithelial inflammatory models, secretion, barrier integrity, repair, and inflammatory signaling should similarly be interpreted together with target expression and direct functional confirmation.

### 4.3. TMEM16A/ANO1 and Chloride-Channel Pharmacology

Transmembrane protein 16A (TMEM16A)/anoctamin-1 (ANO1) exemplifies why ion-channel analyses must distinguish acute channel block from expression-level downregulation and bona fide degradation [109,110,111]. Acute inhibition refers to the rapid suppression of calcium-activated chloride current and is most rigorously demonstrated by electrophysiological recordings or direct chloride-flux assays conducted before changes in protein abundance can occur.

TMEM16A was identified as a calcium-activated chloride channel through convergent molecular cloning and functional studies [112,113,114]. Subsequent reviews and disease-oriented investigations have implicated ANO1/TMEM16A in secretion, smooth muscle physiology, epithelial transport, cancer signaling, and related pathological contexts [115,116]. Collectively, these findings establish ANO1 as an attractive therapeutic target while simultaneously underscoring the need for precise classification of pharmacological mechanisms.

Expression-level downregulation is mechanistically distinct from acute channel inhibition. A compound may reduce ANO1 protein abundance following prolonged exposure by altering transcription, translation, trafficking, or protein stability. In cancer models, ANO1 overexpression has been associated with invasion, prognosis, and growth-factor signaling [117,118]. Accordingly, drug-discovery claims should specify whether a compound blocks conductance, reduces protein abundance, alters localization, or modulates downstream signaling.

**Figure 1:**
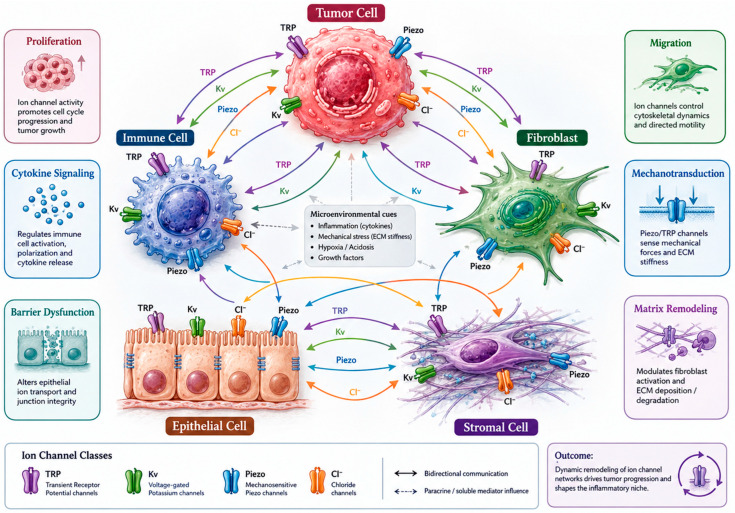
Ion channel network remodeling across cell types. Pathophysiological communication among tumor cells, immune cells, fibroblasts, epithelial barriers, and stromal compartments is organized around TRP, Kv, Piezo, and chloride-channel signaling. The schematic highlights how inflammatory cytokines, mechanical stress, hypoxia/acidosis, growth factors, and extracellular matrix remodeling reshape multicellular ion channel networks in tumor-inflammatory microenvironments.

Other chloride channels and transport pathways provide an important contextual perspective. Bestrophin channels and CFTR-related pathways contribute to bicarbonate and chloride transport, epithelial physiology, and therapeutic development [119,120]. The development of CFTR correctors, such as VX-809, demonstrates that channel and transporter programs may require distinct assays to evaluate function, folding, trafficking, and cellular rescue [121]. For ANO1 and related channels, protein-level modulation therefore cannot be inferred solely from acute functional block. Operationally, acute inhibition and protein-level regulation should be distinguished by concentration, exposure time, normalization strategy, and orthogonal controls. Acute channel inhibition should be assessed over short exposures, typically minutes to less than 1 h, using electrophysiology or direct ion-flux assays at concentrations that do not reduce cell viability or membrane integrity. In contrast, expression-level downregulation or degradation should be evaluated after longer incubations, typically 4–48 h depending on the biological system, using immunoblotting, immunofluorescence, flow cytometry, surface biotinylation, or proteomic approaches. Current inhibition should be normalized to cell capacitance, baseline current density, and recording quality, whereas protein-level changes should be normalized to total protein, loading controls, cell number, viability, and, where appropriate, surface expression. Mechanistic classification is most robust when time-course experiments, washout/reversibility testing, transcriptional analyses, trafficking assays, proteasome or lysosome inhibition, cycloheximide-chase experiments, and genetic rescue or knockdown studies converge on the same interpretation. This multicellular channel-remodeling concept is illustrated in Figure 1.

Representative disease-linked channel classes and their screening implications are summarized in Table 2.

Publicly available reports rarely disclose complete end-to-end ion-channel discovery cascades from docking through organoid or MPS validation, because many such workflows are proprietary. Nevertheless, several well-established examples illustrate why cascade-dependent decision-making is necessary. These examples do not imply universal pass/fail thresholds; rather, they illustrate how potency, mechanism, exposure, selectivity, safety, and disease-context data should be interpreted together. For instance, hERG/Kv11.1 potency should be assessed in relation to unbound exposure and multi-channel effects; Kv1.3 potency should be interpreted together with immune-cell state and KCa3.1 compensation; CFTR modulators require separate assays for folding, trafficking, gating, and epithelial rescue; and TMEM16A/ANO1 pharmacology requires differentiation between acute current block and protein-level regulation and cytotoxicity. Representative examples include CFTR correction and potentiation [122,123,124], hERG/Kv11.1 liability and CiPA-aligned modeling [43,44,45,46,47,48,49,50,51,52,53,54,125], Kv1.3-directed immunomodulation [23,24,105,106,107,109,110,111], TMEM16A/ANO1 pharmacology [112,113,114,115,116,117,118,119,120,121], and MPS exposure/material effects [87,88,89,90,91,92,93,94,126] (Table 3).

## 5. Integrated Hybrid Screening Cascade and Translational Decision Architecture

The following section translates the conceptual framework into a series of practical decision points. Each stage is organized around a specific question that determines whether a compound should progress, be deprioritized, or return for assay redesign or medicinal chemistry optimization. Accordingly, the cascade should be viewed as an iterative decision system rather than as a fixed linear workflow. Each stage answers a distinct pharmacological question. Computational triage asks whether a compound is plausible and developable; optical HTS asks whether it produces scalable functional activity; APC asks whether the activity reflects direct modulation of ionic current; safety profiling asks whether the pharmacology is selective enough to avoid unacceptable electrophysiological liabilities; and organoid or MPS testing asks whether the mechanism remains relevant in a human-like tissue context. Therefore, the value of the cascade lies not in the superiority of any single platform, but in the progressive conversion of weak, indirect, or context-dependent evidence into a coherent translational argument.

### 5.1. Stage 1: Structure and AI-Assisted Triage

The first stage of a hybrid cascade is computational triage. Structural models, docking, ligand similarity, target-family knowledge, and ADMET prediction can reduce a large library to a smaller set of tractable candidates [66,67,68,69,70,71,72,73,74,75,76,77,78,79,80]. In ion-channel programs, this stage should not merely rank docking scores. It should also identify pan-assay interference compounds, reactive chemotypes, likely hERG liabilities, poor solubility, membrane-disruptive structures, and compounds prone to fluorescence interference.

Triage should remain experimentally cautious. A docking pose in a pore, fenestration, voltage-sensor pocket, or lipid-exposed groove is a hypothesis rather than proof of mechanism. Accordingly, Stage 1 should deliver a prioritized, annotated library for functional testing, not a claim of confirmed binding.

### 5.2. Stage 2: Optical HTS and Primary Activity Screening

The second stage is scalable activity screening. Optical HTS can test whether prioritized compounds alter calcium entry, membrane potential, chloride flux, or disease-relevant imaging phenotypes [26,27]. This stage provides an initial functional profile of chemical activity and helps define concentration ranges for follow-up assays.

Primary hits should then proceed through orthogonal counter-screens. For example, a calcium-flux hit should be evaluated for autofluorescence, quenching, cytotoxicity, and effects on upstream receptors or calcium stores [62,63,64,65]. A chloride-flux hit should be assessed for dye artifacts, transporter effects, and changes in cell number. This step is essential because it prevents assay-interference artifacts from advancing through the cascade. At this stage, chemical interference should be treated as a major source of false-positive activity. In addition to autofluorescence, quenching, dye-loading artifacts, transporter effects, and cytotoxicity, screening cascades should consider pan-assay interference compounds, colloidal aggregation, reactive chemotypes, and nonspecific membrane perturbation [127,128]. These liabilities are particularly relevant for ion-channel assays because changes in membrane potential, intracellular calcium, chloride flux, or fluorescence intensity can arise from indirect effects rather than direct channel modulation. Therefore, primary optical hits should be triaged using orthogonal counter-screens, cytotoxicity normalization, dye-free or label-free assays, detergent-sensitivity or aggregation controls when appropriate, and direct electrophysiological confirmation before mechanistic claims are made.

### 5.3. Stage 3: APC Confirmation and State-Aware Pharmacology

The third stage is APC confirmation. Here, hits are evaluated for direct current modulation, concentration dependence, voltage dependence, kinetics, reversibility, and use or state dependence [25,26,27,28,29]. The aim is to determine whether a compound acts as a pore blocker, allosteric modulator, gating modifier, kinetic stabilizer, or indirect perturbant.

State-aware protocols are essential when disease relevance depends on altered excitability or repeated stimulation. In sodium and potassium channels, depolarization frequency and holding potential can change apparent potency. In calcium-activated chloride channels, intracellular calcium, voltage, splice context, and regulatory domains can shape activity. In mechanosensitive channels, stimulus waveform and membrane tension are integral parts of the pharmacological protocol. These parameters should be specified because they determine whether SAR data can be interpreted reliably.

### 5.4. Stage 4: Safety Pharmacology and Attrition Control

The fourth stage is early safety pharmacology. hERG, Nav1.5, Cav, and other cardiac or neuronal channels should be profiled early when chemical features or target class suggest electrophysiological risk [33,43,44,45,46,47,48,49,50,51,52,53,54]. This is particularly important in ion-channel discovery because on-target potency and off-target channel activity often occupy similar physicochemical space.

CiPA-aligned thinking improves safety interpretation. Instead of treating hERG inhibition as a binary stop/go criterion, integrated risk assessment considers multi-channel effects, action-potential simulations, hiPSC-cardiomyocyte behavior, and exposure margins [44,45,46,47,48,49]. For medicinal chemistry, this framework can distinguish compounds that require structural redesign from those whose risk may be considered manageable due to counterbalancing channel effects or low exposure, reducing proarrhythmic liability. An important component of CiPA-aligned interpretation is the translation of in vitro ion-channel inhibition data into in silico human ventricular action-potential models. Multi-channel data can be incorporated into mathematical cardiomyocyte models to estimate how simultaneous effects on IKr, INa, ICa, L, IKs, and other currents influence repolarization reserve, action-potential duration, and torsadogenic risk [44,45,46,47,48,49,129]. This modeling step is particularly valuable because hERG inhibition alone does not always predict clinical proarrhythmia, and some compounds may show counterbalancing effects on inward or outward currents. Therefore, cardiac safety profiling in ion-channel discovery should integrate concentration-dependent channel inhibition, exposure margins, in silico action-potential simulation, and hiPSC-cardiomyocyte assays rather than relying on a single hERG IC50 threshold.

Safety pharmacology for ion-channel modulators should also extend beyond hERG/Kv11.1 and formal proarrhythmia prediction. Depending on the target and chemotype, relevant liabilities may include Nav1.5-associated conduction slowing, Cav1.2-associated effects on cardiac or vascular contractility, neuronal excitability changes, smooth-muscle effects, epithelial secretion or barrier disruption, immune suppression, mitochondrial toxicity, and nonspecific membrane perturbation. Importantly, some liabilities may arise from on-target effects in non-disease tissues rather than from classical off-target activity. Therefore, safety profiling should be selected according to target distribution, disease indication, expected exposure, physicochemical properties, and the therapeutic window required for the intended patient population. Broader safety liabilities relevant to ion-channel modulators are summarized in Table 4.

### 5.5. Stage 5: DMPK, Exposure, and Developability Integration

Drug metabolism and pharmacokinetics (DMPK) and exposure assessment should be integrated into the ion-channel screening cascade rather than treated as a late-stage developability check. Ion-channel modulators are often lipophilic, amphiphilic, or weakly basic molecules, properties that may improve membrane access but can also increase plasma protein binding, tissue accumulation, nonspecific membrane interactions, metabolic liability, and slow clearance. Therefore, apparent potency should be interpreted in relation to unbound exposure, permeability, metabolic stability, transporter liability, and the expected therapeutic window. A compound with attractive electrophysiological potency may still be deprioritized if it exhibits poor solubility, high nonspecific binding, a low free fraction, rapid metabolic turnover, reactive-metabolite formation, excessive tissue accumulation, or a high risk of drug–drug interactions. DMPK and exposure-related decision points for ion-channel modulators are summarized in Table 5.

DMPK considerations are also directly linked to safety pharmacology. For example, hERG/Kv11.1 or Nav1.5 inhibition should be interpreted relative to free plasma concentration, tissue exposure, and predicted clinical dosing rather than as an isolated IC50 value. Similarly, organoid or MPS inactivity may reflect insufficient free exposure, protein binding, matrix binding, poor penetration, or absorption by device materials rather than the absence of target relevance. Thus, DMPK data should inform compound prioritization at multiple stages: early chemical triage, APC concentration selection, safety-margin estimation, organoid/MPS exposure interpretation, and candidate nomination.

### 5.6. Stage 6: Organoid and MPS Translational Validation

The sixth stage tests whether a mechanistically confirmed modulator alters a disease-relevant phenotype in a human-context model. Patient-derived tumor organoids can be used to assess whether channel modulation affects viability, invasion, differentiation, or treatment response [60,61,82,83,84]. Epithelial organoids can assess barrier function, secretion, repair, and inflammatory responses. These assays are most informative when the measured phenotype is explicitly linked to channel mechanisms.

MPS platforms add flow, stretch, shear stress, compartmentalization, and tissue–tissue interfaces [85,86,87,88,89,90,91,92]. For mechanosensitive or barrier-associated channels, these physical variables may be essential rather than optional. A modulator that fails in a static two-dimensional assay may become relevant under mechanical load, while a potent recombinant blocker may fail in MPS due to limited exposure, protein binding, or poor tissue penetration. A practical limitation of MPS platforms is that device materials can alter compound exposure. Polydimethylsiloxane (PDMS), which has been widely used in microfluidic devices because of its transparency, gas permeability, and ease of fabrication, can absorb hydrophobic small molecules and thereby reduce the free concentration available to cells [130]. This issue is particularly relevant for ion-channel modulators, many of which are lipophilic and membrane-associated. As a result, a compound that is active in electrophysiological assays may appear weak or inactive in an MPS device if its effective exposure is reduced by material absorption. Whenever possible, MPS studies should therefore consider compound recovery, free-fraction measurement, material controls, alternative device materials, or exposure correction before negative results are interpreted as a lack of biological activity.

### 5.7. Stage 7: Candidate Selection and Evidence Integration

The final stage integrates potency, selectivity, safety, exposure, mechanism, and disease-model efficacy into a candidate-selection decision. A credible ion-channel lead should show orthogonal functional activity, direct current modulation, interpretable kinetics, acceptable selectivity, a manageable safety margin, and evidence of disease-context activity. No single assay can provide all of this information.

**Figure 2:**
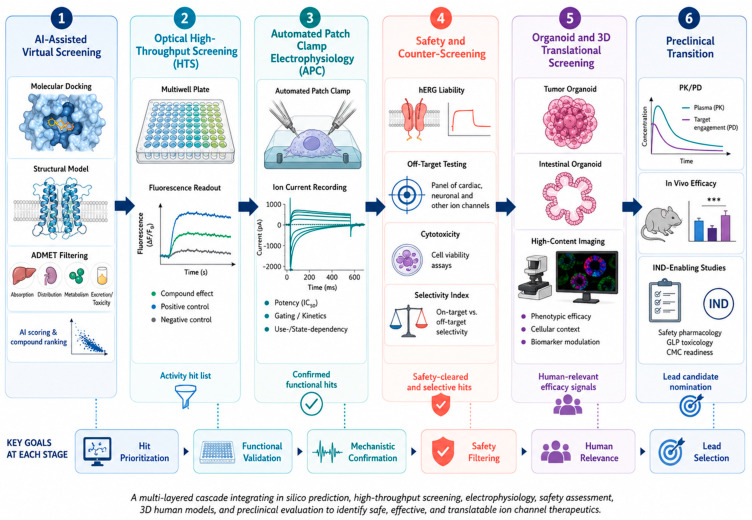
Hybrid screening cascade workflow for ion-channel drug discovery. The workflow integrates artificial intelligence (AI)-assisted virtual screening, optical high-throughput screening (HTS), automated patch-clamp (APC) electrophysiology, safety and counter-screening, organoid or three-dimensional translational models, and preclinical transition. Each stage contributes a distinct decision layer. AI-assisted triage prioritizes plausible and developable chemotypes; optical HTS identifies scalable functional activity while excluding assay-interference artifacts; APC confirms direct current modulation, kinetic behavior, reversibility, and state-dependent pharmacology; safety profiling evaluates hERG/Kv11.1, voltage-gated sodium channel Nav1.5, voltage-gated calcium (Cav) channels, cytotoxicity, and off-target liabilities; organoid and microphysiological system (MPS) models test whether the mechanism retains relevance in human-like disease contexts; and preclinical transition integrates pharmacokinetics/pharmacodynamics (PK/PD), efficacy, safety, target engagement, and candidate-readiness criteria. Feedback loops indicate that safety liabilities may redirect medicinal chemistry, APC artifacts may require revised optical protocols, and organoid or MPS failure may prompt exposure, penetration, or target-engagement reassessment. The cascade emphasizes staged evidence accumulation rather than reliance on single-assay potency as the basis for lead selection. The asterisks (***) indicate an illustrative statistical significance marker in the schematic efficacy inset.

This decision architecture should also be iterative. Safety findings may send a compound back to medicinal chemistry, organoid failure may trigger exposure or target-engagement studies, and APC results may require revised optical protocols. The strength of a hybrid cascade lies not in its linearity but in its explicit, evidence-based decision points. The proposed staged decision architecture is summarized schematically in Figure 2.

**Figure 3:**
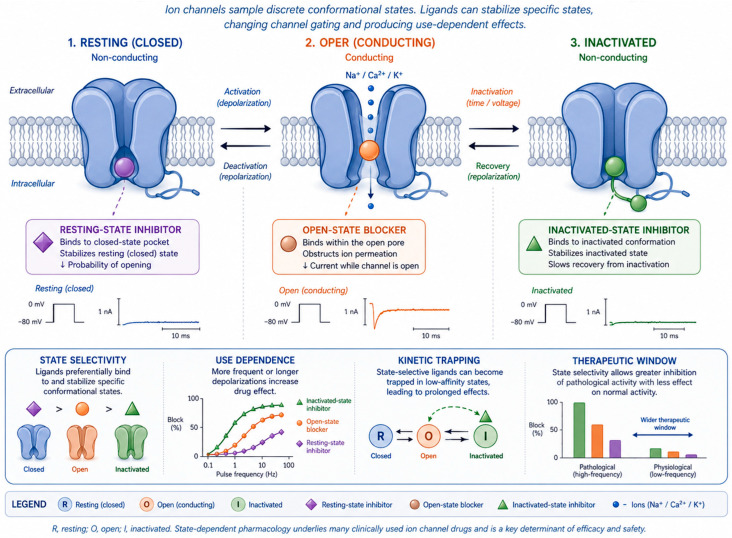
State-dependent ion-channel pharmacology. Ion channels cycle among resting, open, and inactivated conformations. Ligands may preferentially stabilize specific states, producing resting-state inhibition, open-state block, inactivated-state inhibition, use dependence, kinetic trapping, and disease-state selectivity. Examples include use-dependent sodium-channel block by local anesthetics or antiarrhythmic agents, as well as protocol-dependent modulation of potassium and calcium channels. These properties explain why voltage protocols and kinetic analyses are central to ion-channel pharmacology. Colors indicate state classes and corresponding inhibitor types: purple, resting/closed state; orange, open/conducting state; and green, inactivated state.

The pharmacological importance of channel-state occupancy is illustrated in Figure 3.

**Figure 4:**
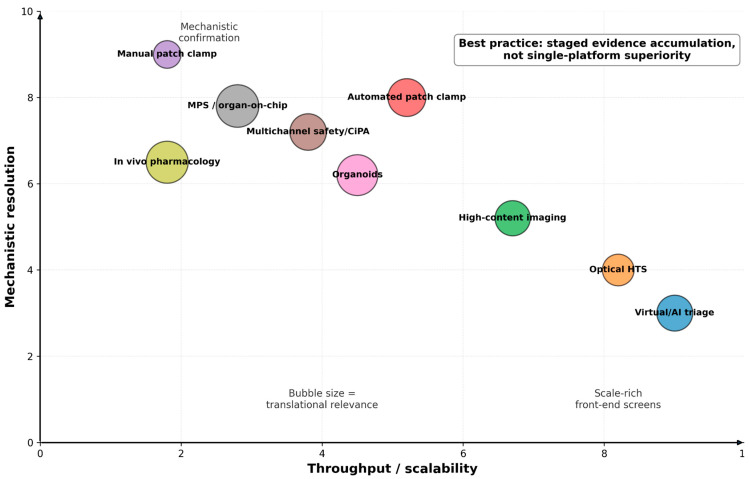
Qualitative evidence map of ion-channel discovery platforms. Platforms are positioned according to two conceptual axes: throughput/scalability and mechanistic resolution. Bubble size represents approximate translational relevance, defined by the extent to which a platform captures disease-relevant cellular context, safety-relevant physiology, or human tissue complexity. The map is intended as a heuristic framework rather than a quantitative ranking system. Its purpose is to illustrate complementarity among platforms: high-throughput approaches are efficient for front-end discovery, electrophysiological methods provide direct mechanistic confirmation, safety panels reduce attrition risk, and organoid/MPS or in vivo models increase translational confidence. The key message is staged evidence accumulation, not single-platform superiority. Practical criteria for distinguishing acute channel inhibition from protein-level channel remodeling are summarized in Table 6.

A qualitative comparison of screening platforms according to throughput, mechanistic resolution, and translational relevance is shown in Figure 4.

**Table 6:** Practical criteria for distinguishing acute channel inhibition from protein-level channel remodeling.

Mechanistic Category	Typical Experimental Window	Primary Readout	Key Normalization	Essential Controls
Acute channel inhibition	Minutes to less than 1 h	Patch-clamp current, current density, and direct ion-flux or voltage readouts	Baseline current, cell capacitance, seal quality, recording stability, non-cytotoxic concentration	Vehicle control, washout/reversibility, viability, membrane integrity, off-target channel or pathway counterscreen
State- or use-dependent block	Seconds to minutes during defined stimulation protocols	Voltage-, ligand-, frequency-, or use-dependent changes in current	Holding potential, pulse frequency, recovery interval, current rundown correction	Protocol variation, recovery analysis, repeated-pulse control, rundown control
Expression-level downregulation	Several hours to days	Total protein abundance, mRNA, immunofluorescence, flow cytometry	Total protein, housekeeping markers, cell number, viability, passage and differentiation state	qPCR, immunoblotting, imaging, non-cytotoxic exposure, transcriptional control
Altered trafficking or localization	Hours to days	Surface protein abundance, subcellular localization, and membrane abundance	Surface/total protein ratio, membrane marker, cell number, viability	Surface biotinylation, confocal imaging, trafficking marker, washout or rescue
Degradation	Hours to days	Protein half-life and degradation-pathway markers	Loading controls, total protein, cell viability	Cycloheximide chase, proteasome/lysosome inhibitor rescue, ubiquitination or degradation marker analysis
Indirect phenotypic effect	Variable	Viability, migration, invasion, cytokine output, organoid growth	Cell number, viability, exposure, target expression	Genetic knockdown/rescue, orthogonal channel assay, cytotoxicity and mitochondrial controls

### 5.8. Practical Decision Points in an Integrated Ion-Channel Screening Cascade

A practical cascade should define not only which platforms are used, but also what decision each platform supports. Computational triage should generate a prioritized and annotated library, not a claim of confirmed binding. Optical HTS should identify reproducible functional activity while excluding assay interference. APC should determine whether the compound directly modulates ionic current under defined voltage, ligand, mechanical, or calcium-dependent protocols. Safety and DMPK profiling should define whether on-target and off-target channel activities are compatible with the intended exposure and therapeutic window. Organoid and MPS assays should test whether the mechanism remains relevant in a human-like tissue context while controlling for exposure, viability, penetration, and model heterogeneity. Discordant results should not automatically terminate a program; rather, they should trigger predefined feedback actions such as assay redesign, medicinal chemistry optimization, exposure analysis, genetic validation, or target-engagement studies.

## 6. Limitations and Future Directions

The proposed framework has several limitations. First, AI- and structure-enabled methods depend on training data, model quality, conformational sampling, and assumptions about ligand-binding states [66,67,68,69]. In ion channels, lipid interactions, auxiliary subunits, splice variants, glycosylation, phosphorylation, and voltage-dependent conformational changes can all influence ligand recognition. Static models cannot fully capture this complexity.

Second, optical HTS can overestimate activity when compounds interfere with fluorescent dyes, compromise cell health, perturb intracellular stores, or alter upstream signaling [62,63,64,65]. Reports should therefore include assay-quality metrics, concentration–response behavior, and orthogonal confirmation. Without these controls, an apparently strong high-throughput signal may not reflect direct channel pharmacology.

Third, APC is mechanistically powerful but still prone to artifacts. Seal quality, cell-line background currents, series resistance, rundown, voltage-control limits, and protocol design can influence apparent potency. These issues are manageable, but they require transparent reporting of voltage protocols, stimulus timing, holding potentials, compound exposure, and acceptance criteria.

Fourth, organoid and MPS models improve translational relevance but also introduce variability. Matrix composition, passage number, tissue source, differentiation state, immune components, vascularization, and mechanical inputs can alter channel expression and drug response [55,56,57,58,59,60,61,82,83,84,85,86,87,88,89,90,91,92,93,94,95]. Standardization is improving, but these platforms are best used as defined translational filters rather than universal replacement assays.

Future directions include closed-loop systems that link AI-generated hypotheses to APC confirmation and organoid or MPS phenotyping. Patient-derived organoids, engineered immune co-cultures, microfluidic mechanical stimulation, and digital-twin pharmacology may enable indication-specific selection of channel modulators. The next major advance will likely arise from integrating temporal electrophysiology, spatial cell biology, and human-context disease models, rather than from further optimization of any single assay in isolation [122,123,124,125,126]. Recent advances in structural prediction may further support hypothesis generation for ion-channel complexes and ligand-accessibility questions. For example, AlphaFold 3 extends structure prediction beyond single proteins toward biomolecular complexes containing proteins, nucleic acids, small molecules, ions, and modified residues [131]. Nevertheless, in ion-channel drug discovery, such predictions are most useful when treated as experimentally testable hypotheses and integrated with electrophysiological, kinetic, safety, DMPK, and disease-context validation.

## 7. Conclusions

Ion channels are validated but challenging drug targets. Their discovery is complicated by dynamic gating, state dependence, subtype similarity, membrane context, disease-specific remodeling, and safety-relevant off-target effects [1,2,3,4,5,6,7]. These features explain why ion-channel pharmacology cannot be defined by potency values from a single assay alone.

A modern ion-channel program should begin with structure-informed and AI-assisted prioritization, advance through scalable optical screening, confirm mechanism with APC current recordings, de-risk off-target electrophysiology early, and test disease relevance in human-like organoid or MPSs [25,26,27,28,29]. This staged workflow ensures that each assay answers a specific question.

This hybrid cascade is particularly important in cancer and inflammatory disease, where ion-channel function is reshaped by microenvironmental stress, immune activation, epithelial polarization, mechanical input, and therapeutic pressure [20,21,22,23,24,34,35,36,37,39,40,41,42,96,97,98,99,100,103,104,105,106,107]. These disease contexts illustrate why ion-channel pharmacology cannot be reduced to potency values from a single assay. Selective and translatable ion-channel therapeutics require integrated evidence across computational prediction, functional screening, electrophysiological confirmation, safety pharmacology, and human-relevant disease models. The next generation of ion-channel therapeutics will likely emerge from disciplined decision cascades that connect state-aware pharmacology with disease-context biology.

## Figures and Tables

**Table 1:** Comparative roles of screening modalities in an integrated ion channel discovery cascade.

Modality	Primary Value	Key Readout	Main Limitation	Best Use
AI/In Silico triage	Library enrichment and liability filtering	Docking, structure models, ADMET, chemotype prioritization	Model bias; static or incomplete conformations	Early prioritization before wet-lab testing
Optical HTS	Scalable activity discovery	Calcium, voltage, chloride, imaging, label-free signals	Indirect readout; fluorescent interference	Primary screening and concentration-response triage
APC electrophysiology	Mechanistic confirmation	Current amplitude, kinetics, voltage dependence, state dependence	Cost, throughput, cell-line dependence	Hit validation, SAR, mechanism assignment
Safety panels	Attrition control	hERG, Nav1.5, Cav, CiPA, hiPSC-CM	Panel selection and exposure assumptions	Early cardiac and off-target de-risking
Organoids/MPSs	Human-context translation	Phenotype, biomarkers, viability, barrier function, tissue response	Variability and standardization	Preclinical prioritization after mechanism is defined

**Table 2:** Disease-linked channel classes and translational implications.

Channel Class	Disease Linkage	Screening Implication	Representative Refs.
TRP channels	Calcium entry, stress sensing, migration, inflammation, pain	Optical calcium HTS followed by APC or orthogonal functional confirmation	[20,34,35,36,37]
Kv1.3/K+ channels	T-cell activation, autoimmunity, proliferation, immune-state selectivity	Voltage protocols, immune-cell assays, selectivity panels	[23,24,103,104,105,106,107]
Piezo channels	Mechanotransduction, stromal tension, vascular biology, migration	Mechanical stimulation or chemical activation plus direct functional readouts	[96,97,98,99,100,101,102]
TMEM16A/chloride channels	Epithelial transport, secretion, tumor signaling, protein-level regulation	Flux assays, APC, protein-abundance and degradation validation	[109,110,111,112,113,114,115,116,117,118]
CFTR/bestrophin-related pathways	Epithelial chloride/bicarbonate transport and rescue pharmacology	Separate function, folding, trafficking, and rescue assays	[119,120,121]

**Table 3:** Examples of cascade-dependent decision points in ion-channel drug discovery.

Example	Why a Single Assay Can Mislead	Cascade-Dependent Decision Point	Practical Interpretation
hERG/Kv11.1 liability and CiPA-aligned assessment	A single hERG IC50 value may overestimate or underestimate clinical proarrhythmic risk because torsadogenic liability depends on unbound exposure, multi-channel effects, and repolarization reserve.	hERG inhibition should be interpreted together with Nav1.5, Cav1.2, IKs and other cardiac currents, in silico ventricular action-potential modeling, hiPSC-CM assays, and exposure margins [43,44,45,46,47,48,49,50,51,52,53,54,125].	A compound should not be advanced or discontinued solely on the basis of hERG potency. The ratio between hERG IC50 and free therapeutic exposure, together with multi-channel balance, should guide the decision.
Kv1.3-directed immunomodulation	Potent Kv1.3 inhibition in a recombinant or simplified system may not predict activity in disease-relevant immune cells if T-cell state, KCa3.1 compensation, selectivity, delivery, or exposure are not considered.	APC should be combined with activation-state-defined T-cell assays, cytokine readouts, selectivity profiling, and pharmacokinetic feasibility [23,24,105,106,107,109,110,111].	A Kv1.3 hit should progress only if channel inhibition occurs at achievable, non-cytotoxic exposures and translates into functional modulation of immune cells in the relevant activation state.
CFTR correction and potentiation	A single chloride-flux or rescue endpoint cannot distinguish improved folding, trafficking, gating, membrane stability, or downstream epithelial rescue.	Folding, trafficking, surface localization, gating, chloride transport, and epithelial rescue should be evaluated using complementary assays [122,123,124]	Channel and transporter programs may require mechanism-specific assays rather than a single functional endpoint.
TMEM16A/ANO1 pharmacology	Reduced chloride current, reduced protein abundance, cytotoxicity, altered calcium signaling, and off-target pathway modulation can generate similar phenotypes.	Acute current inhibition should be separated from expression-level downregulation, altered localization, degradation, and nonspecific cytotoxicity [112,113,114,115,116,117,118,119,120,121].	Claims of ANO1 inhibition should specify whether the compound blocks conductance, reduces protein abundance, alters localization, or indirectly modulates cell signaling.
MPS exposure and material effects	A compound that is active in APC may appear inactive in an MPS device because of limited penetration, nonspecific binding, or absorption by device materials.	MPS results should be interpreted with exposure controls, free-fraction assessment, compound recovery, material compatibility checks, and viability normalization [87,88,89,90,91,92,93,94,126].	A negative MPS result should prompt exposure and penetration analyses before concluding that the mechanism lacks disease relevance.

**Table 4:** Broader safety liabilities to consider for ion-channel modulators.

Safety Domain	Representative Concern	Suggested de-Risking Approach
Cardiac repolarization	hERG/Kv11.1 inhibition, delayed repolarization, torsadogenic risk	hERG assay, multi-ion-channel panel, in silico action-potential modeling, hiPSC-CM assays, exposure-margin analysis
Cardiac conduction	Nav1.5 inhibition and conduction slowing	Nav1.5 profiling, use-dependent block assessment, QRS-related risk consideration
Contractility and vascular tone	Cav1.2 or smooth-muscle channel effects	Cav profiling, contractility assays, vascular or smooth-muscle functional models
Neuronal excitability	CNS or peripheral nerve hyperexcitability or suppression	Neuronal channel panels, excitability assays, seizure-liability or sensory-neuron models when relevant
Epithelial and secretory function	Altered chloride, calcium, or potassium conductance affecting secretion or barrier integrity	Epithelial barrier, secretion, transepithelial resistance, and viability assays
Immune function	Excessive immunosuppression or dysregulated cytokine signaling	Immune-cell activation assays, cytokine profiling, cell-state-specific selectivity analysis
Nonspecific membrane or mitochondrial toxicity	Apparent channel inhibition due to membrane disruption, mitochondrial stress, or cytotoxicity	Cytotoxicity assays, mitochondrial counterscreens, detergent/aggregation controls, orthogonal electrophysiology

**Table 5:** DMPK and exposure-related decision points for ion-channel modulators.

DMPK Parameter	Why It Matters for Ion-Channel Modulators	Suggested Decision Use
Solubility and aggregation risk	Poor solubility or colloidal aggregation can mimic channel inhibition or distort concentration–response curves.	Use solubility limits, detergent/aggregation controls, and orthogonal assays during hit triage.
Permeability and transporter liability	Intracellular binding sites or tissue models may require adequate cellular penetration, while efflux transporters may reduce exposure.	Interpret optical, APC, organoid, and MPS potency together with permeability and transporter data.
Plasma protein binding and free fraction	Highly lipophilic compounds may show strong protein binding, making total concentration misleading.	Compare potency and safety liabilities with unbound rather than total exposure.
Metabolic stability and clearance	Rapid clearance may prevent target engagement, whereas slow clearance may increase accumulation and safety risk.	Use metabolic stability and clearance data to prioritize compounds before advanced translational models.
Tissue distribution and accumulation	Membrane-associated compounds may accumulate in cardiac, neuronal, or epithelial tissues.	Evaluate tissue exposure when interpreting on-target and off-target ion-channel effects.
Drug–drug interaction risk	CYP inhibition, transporter interactions, or altered clearance may increase exposure and safety liabilities.	Include DDI risk in candidate nomination, especially for chronic disease indications.
Exposure in organoids/MPS	Matrix binding, protein binding, limited penetration, and device-material absorption can reduce free concentration.	Measure or estimate free exposure before interpreting negative results from translational models.

## Data Availability

No new data were created or analyzed in this review. Data sharing is not applicable to this article.

## Abbreviations

The following abbreviations are used in this manuscript:

ADMET	Absorption, distribution, metabolism, excretion, and toxicity
ANO1	anoctamin-1
APC	automated patch-clamp
CiPA	Comprehensive in Vitro Proarrhythmia Assay
hERG	human Ether-à-go-go-Related Gene
HTS	high-throughput screening
MPS	microphysiological system
TMEM16A	transmembrane protein 16A
TRP	transient receptor potential
